# Desired Turbulence? Gut-Lung Axis, Immunity, and Lung Cancer

**DOI:** 10.1155/2017/5035371

**Published:** 2017-09-17

**Authors:** Rea Bingula, Marc Filaire, Nina Radosevic-Robin, Mathieu Bey, Jean-Yves Berthon, Annick Bernalier-Donadille, Marie-Paule Vasson, Edith Filaire

**Affiliations:** ^1^University of Clermont-Auvergne, UMR 1019 INRA-UCA, Human Nutrition Unit (UNH), ECREIN Team, 63000 Clermont-Ferrand, France; ^2^Jean Perrin Comprehensive Cancer Centre, Thoracic Surgery Unit, 63011 Clermont-Ferrand, France; ^3^University of Clermont-Auvergne, Jean Perrin Comprehensive Cancer Centre, Department of Pathology, INSERM U1240 Molecular Imaging and Theranostic Strategies, 63000 Clermont-Ferrand, France; ^4^Greentech SA, Biopôle Clermont-Limagne, 63360 Saint-Beauzire, France; ^5^INRA, UR454 Microbiology Unit, Clermont-Ferrand/Theix Research Centre, 63122 Saint-Genès-Champanelle, France; ^6^Jean Perrin Comprehensive Cancer Centre, CHU Gabriel-Montpied, Human Nutrition Unit (UNH), CRNH Auvergne, 63000 Clermont-Ferrand, France; ^7^CIAMS, University Paris-Sud, University Paris-Saclay, 91405 Orsay Cedex, France; ^8^CIAMS, University of Orléans, 45067 Orléans, France

## Abstract

The microbiota includes different microorganisms consisting of bacteria, fungi, viruses, and protozoa distributed over many human body surfaces including the skin, vagina, gut, and airways, with the highest density found in the intestine. The gut microbiota strongly influences our metabolic, endocrine, and immune systems, as well as both the peripheral and central nervous systems. Recently, a dialogue between the gut and lung microbiota has been discovered, suggesting that changes in one compartment could impact the other compartment, whether in relation to microbial composition or function. Further, this bidirectional axis is evidenced in an, either beneficial or malignant, altered immune response in one compartment following changes in the other compartment. Stimulation of the immune system arises from the microbial cells themselves, but also from their metabolites. It can be either direct or mediated by stimulated immune cells in one site impacting the other site. Additionally, this interaction may lead to immunological boost, assisting the innate immune system in its antitumour response. Thus, this review offers an insight into the composition of these sites, the gut and the lung, their role in shaping the immune system, and, finally, their role in the response to lung cancer.

## 1. Introduction

The microbiota is a consortium of different microorganisms that includes bacteria (microbiota), fungi (mycobiota), viruses, and protozoa [1] residing on the skin, and in the oral, pulmonary, urogenital, and gastrointestinal (GI) cavities—with the gastrointestinal having the highest density of microbiota. Weighing approximately 1.5 kg, microbial residents in the GI tract outnumber human cells 10-fold and genome size 100-fold. Their functional importance for the host is undeniable involving functions that range from breaking down complex dietary polysaccharides [2] to competing with pathogens and modulating the mucosal and immune system in general [3]. Gut dysbiosis is now considered to be an underlying cause for a wide range of GI diseases and an emerging number of non-GI conditions such as obesity and cardiovascular disease, as well as a range of psychiatric diseases [4, 5]. Recent studies have also reported the effects that the intestinal microbiota exerts on the lungs. This has been referred to as the “gut-lung axis,” which in most cases is mediated by inflammation involving the translocation of bacteria and bacterial products across the GI tract barrier and into blood vessels [6]. However, data on this topic is scarce. Studies of the lung microbiota and its interconnection with other systems and organisms are an emerging field, which is rapidly accumulating evidence to demonstrate that the lungs are not in fact sterile but contain distinct microbial communities [7]. Moreover, it appears that chronic lung diseases such as cancer are linked to a dysbiotic airway microbiota and commonly occur alongside GI disorders [8, 9]. Likewise, individuals with irritable bowel syndrome sometimes have impaired lung function [10]. This leads us to the conclusion that the axis between lung and gut can be considered bidirectional.

In this review, we give an overview of the composition of both the gut and the lung and describe the interaction between the immune system and microbiota using the intestinal tract as an example. In the case of the lungs, we are still only able to speculate about any similarity. We also examine immune stimulation of the gut to observe the effects on lung immunity, inflammation, and lung cancer, and finally, we discuss how these two sites might “cooperate” to achieve a productive immune and anticancer response.

## 2. Gut Microbiota

The evolution of an individual's microbiota begins at birth, with its composition becoming relatively stable after the age of two and remaining so throughout life. The GI tract is populated by more than 1,000 bacterial species. At the level of the phylum, the composition of the microbiota is similar in most healthy people. Over 90% of bacterial cells are Firmicutes and Bacteroidetes, followed by Actinobacteria, Proteobacteria, and Verrucomicrobia, together constituting 99% of the overall commensal microbiota [11]. Around 60 species have been identified as the “core” microbiota, mostly bacteria from the* Bacteroides, Bifidobacterium, Eubacterium, Ruminococcus, *and* Faecalibacterium* genera, as well as a few others [11]. A summary overview of the most prevalent human GI microbiota is shown in Table 1.

Strongly correlating to long-term diet [24], different enterotypes can also be identified through a variation in the levels of one of the three most abundant genera:* Bacteroides *(enterotype 1),* Prevotella* (enterotype 2), and* Ruminococcus* (enterotype 3), which is often less clearly distinguished [25].

### 2.1. The Impact of the GI Microbiota on Host Mucosal Immunity

**Figure 1:**
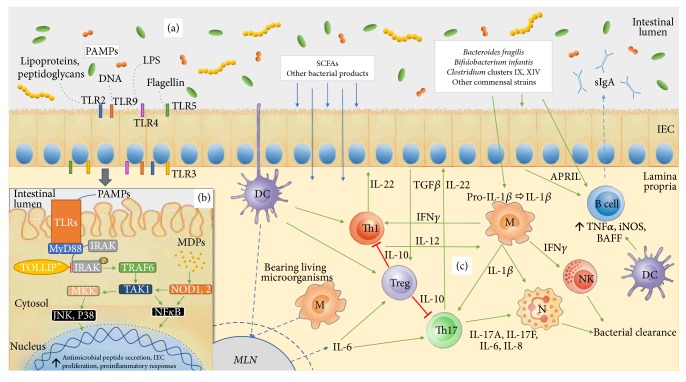
Interaction of the microbiota and intestinal mucosa. (a) Microorganisms in the intestine provide pathogen-associated molecular patterns (PAMPs) that serve as ligands for different Toll-like receptors (TLRs) on the luminal or basolateral surface of the intestinal epithelial cells (IECs). (b) TLRs stimulation activates a signalling cascade resulting in transcription factor activation and gene transcription, enhancing the cell barrier and further stimulating the immunological cells in the lamina propria. This cascade can be inhibited by toll-interacting protein (TOLLIP). ^*∗*^Inhibition seen only for TLR2 and TLR4 [23]. (c) Commensal bacteria and their derivatives (e.g., short-chain fatty acids (SCFAs)) directly stimulate IECs (b) or can be phagocytosed by DCs and macrophages in lamina propria and carried to mesenteric lymph nodes (MLN) where they prime naïve B and T cells to mature and differentiate. B cells become plasma cells and produce IgA that is secreted into the intestinal lumen (sIgA). T cells profile into Th17 and Th1, with proinflammatory tendency, activating additional effector cells as neutrophils, resulting in bacterial clearance. There is also differentiation to Treg cells having anti-inflammatory properties and controlling inflammation. APRIL: a proliferation-inducing ligand; BAFF: B cell-activating factor of the tumour necrosis family; DC: dendritic cell; IEC: intestinal epithelial cells; IFN: interferon; IL: interleukin; iNOS: inducible nitric oxide synthase; IRAK: interleukin receptor-associated kinase; JNK: c-Jun N-terminal kinases; LLN: lung lymph node; LPS: lipopolysaccharide; M: macrophage; MDPs: microbiota derived particles; MKK: mitogen-activated protein kinase kinase; MLN: mesenteric lymph node; MyD88: myeloid differentiation primary response gene 88; N: neutrophil; NF-*κ*B: nuclear factor kappa-light-chain-enhancer of activated B cells; NK: natural killer cell; NOD: nucleotide-binding receptor; PAMPs: pathogen-associated molecular patterns; SCFAs: short-chain fatty acids; TAK-1: transforming growth factor beta-activated kinase; TGF*β*: transforming growth factor beta; Th: T-helper cell; TLR: toll-like receptor; TNF*α*: tumour necrosis factor alfa; TOLLIP: toll-interacting protein; TRAF6: TNF receptor-associated factor 6; Treg: regulatory T cell.

The microbiota is now considered key to the proper development, maturation, and reactivity of the immune system [6, 26]. Microorganisms serve as an inexhaustible source of microorganism-associated molecular patterns (MAMPs) as well as pathogen-associated molecular patterns (PAMPs). The two are recognizable on the host's cells through pattern-recognition receptors (PRRs), which include toll-like receptors (TLRs) and nucleotide-binding receptors (NODs) [27]. TLRs are conserved receptors of the innate immune system that recognize MAMPs and PAMPs among other molecules, evoking different immunological reactions depending on the type of the cell, ligand, and the receptor itself (some of the most common pairs are shown in Figure 1(a)). TLRs, which are in direct contact with the intestinal lumen, are found not only in intestinal epithelial cells (IECs) but also on immune cells within the lamina propria, such as macrophages, dendritic cells (DCs), B cells, T cells, and stromal cells. In IECs, TLR activation by microbial ligands results in epithelial cell proliferation and the expression of antimicrobial peptides, and secretion of immunoglobulin A (IgA) produced by plasma cells in lamina propria, into the gut lumen [23], as well as the expression of antimicrobial peptides. All of the above lead to enhanced intestinal barrier function and limit the possibility of microbial breach. Interestingly, some of the TLRs, such as TLR2 and TLR4, are inhibited by IEC's intracellular Toll-interacting protein (TOLLIP) (Figure 1(b)) when the signal comes from the intestinal lumen, which suggests a selective inflammatory response reserved to microbes that have breached the intestinal barrier [23]. NOD-like receptors, or nucleotide-binding domain, leucine-rich repeat containing proteins (NLRs), are cytoplasmatic equivalents of TLRs that detect bacterial PAMPs entering the mammalian cell (Figure 1(b)). They are especially important in tissues where TLRs are expressed at low levels, for example, in GI tract epithelial cells where the cells are in constant contact with the microbiota, and the expression of TLRs must be downregulated to avoid overstimulation [28].

Commensal microorganisms can enter intestinal lamina propria in several ways: through an opening in the barrier as a result of injury or through active sampling by DCs or M cells. In any case, microorganisms in the lamina propria are either phagocytosed and eliminated by macrophages [29] or engulfed by DCs (along with B cells, both are considered the “professional” antigen presenting cells (APCs)) and are carried live to the mesenteric lymph nodes (Figure 1(c)). Recognition of infected apoptotic cells and bacteria results in the upregulation of interleukin 6 (IL-6), which drives the differentiation of proinflammatory T-helper-IL-17-producing (Th17) cells. Th17 cells primarily produce two main members of the IL-17 family, IL-17A and IL-17F which are involved in the recruitment, activation, and migration of neutrophils [30], with granulocytes playing an important role in bacterial clearance. Commensal-bearing DCs also induce the production of protective secretory immunoglobulin A (sIgA) in activated B cells (Figure 1(c)) [31], that is, plasma cells. This sIgA is then distributed across all mucosal surfaces through the recirculation of activated B and T cells. Commensal bacteria also directly promote the expression of factors involved in the induction of IgA^+^ B cells (Figure 1(c)) [32], their survival, and, interestingly, production of the more resistant form of sIgA, exerting its stability and antimicrobial properties [33]. Through this constant “priming” across several layers, microbiota maintains the immune system's readiness and reactivity, making it more capable of a quick and effective response when needed.

Specific populations of commensal bacteria, for example,* Bacteroides fragilis, Bifidobacterium infantis, *and* Clostridium *clusters IV and XIVa, also induce the so-called regulatory T cells (Tregs), a subset of forkhead box P3 positive (FoxP3^+^) CD4^+^ T-helper lymphocytes that maintain gut homeostasis by stimulating the production of anti-inflammatory cytokine IL-10 [34]. These cells serve as a kind of counterbalance to Th17 response, controlling the scope of its reaction and proinflammatory cytokine production. Thus, depletion of Tregs leads to the abnormal expansion of CD4^+^ Th cells, resulting in robust IL-17 and interferon gamma (IFN*γ*) responses in the colonic lamina propria, to produce intestinal inflammation.

To understand more precisely which commensal microbiota has this immunostimulatory effect, the well-known* Bifidobacterium* probiotic group was tested for its ability to induce the full maturation of human peripheral blood mononuclear cells (PBMCs) into DCs. Twelve* Bifidobacterium *strains descending from 4 probiotic species (*Bifidobacterium longum, B. breve, B. bifidum, *and* B. animalis *subsp.* lactis*) were tested, and each was induced to full maturation into DCs but using different Th preferences (Th1 or Th17), depending on the type of* Bifidobacterium *strain with which they were incubated. Also, cell-free culture supernatants were poor inducers of maturation [35], leading us to conclude that live bacterial cells are necessary to induce efficient maturation and antigen presentation in DCs with this type of bacteria.

Likewise, introducing probiotic strains such as* Bifidobacterium lactis *into healthy elderly volunteers with fully developed immune systems resulted in a significant increase in the proportion of mononuclear leukocytes, the phagocytic capacity of mononuclear and polymorphonuclear phagocytes, and the tumoricidal activity of NK cells [36], highlighting the importance of this specific immune-boosting species whose presence directly impacts immune status.

Immunity and inflammation are not necessarily affected by bacterial cells but may be influenced by bacterial products. Bacterial products that have a significant effect on overall host status surely included short-chain fatty acids (SCFAs), by-products of the microbial fermentation of dietary fibre. Among others,* Bacteroides, Bifidobacterium, Propionibacterium, Eubacterium, Lactobacillus, Clostridium, Roseburia, *and* Prevotella *are all remarkable SCFA producers [37]. Fermentation and SCFA production are thought to inhibit the growth of pathogenic organisms by reducing luminal pH [38]. The most abundant SCFAs are acetate, propionate (found mostly in the small and large intestines), and butyrate (found mostly in the caecum and colon), which are primarily derived from carbohydrates [39]. SCFAs have their specific receptors both on leukocytes and on endothelial cells. Known as two formerly orphan G protein-coupled receptors, GPR41 is found in a wide range of tissues including neutrophils, while GPR43 is shown to be highly expressed in immune cells [40]. GPR109a, the third receptor found in the colon epithelium and immune cells, is butyrate-specific and closely associated with the anti-inflammatory effect [41]. Butyrate is one of the most important SCFAs with members of the Firmicutes phylum as major butyrate producers, harbouring genes for the acetyl-CoA pathways. Other than as the main energy source for the intestinal epithelium and its role in barrier integrity [42, 43] following selective transport into the colon epithelium, butyrate manifests broad anti-inflammatory activities such as immune cell activation, proliferation, migration, adhesion, cytokine expression, and cancer cell apoptosis [26, 41]. These are mostly attributed to its function as a histone deacetylase (HDAC) inhibitor. HDAC inhibition influences the acetylation not only of histones (FoxP3 locus in Tregs, important for their maturation) [44], but also of major transcription factors such as nuclear factor kappa-light-chain-enhancer for activated B cells (NF-*κ*B) or signal transducer and activator of transcription 3 (Stat3) [45, 46], major proinflammatory pathway factors affecting the proinflammatory cytokine secretion profile in immune cells [47] and decreasing the proliferation and apoptosis of tumour cells [48].

As mentioned previously, changes to the gut microbiota are related to, for example, changes in diet, antibiotic administration, chemotherapy, and a person's general immune status. Whether with a transient or permanent effect, these changes often lead to dysbiosis, with an altered ratio of beneficial bacterial species (*Lactobacillus *sp.*, Faecalibacterium prausnitzii*, etc.) and/or an overgrowth or population shift of other species [26]. In this event, expanded indigenous microorganisms (now potential opportunistic pathogens) may also produce DNA-damaging superoxide radicals and genotoxins in significant concentrations and induce innate immune mediated proinflammatory pathways [49], directly damaging cells and promoting malignant transformation by inducing chromosomal and microsatellite instability, CpG island methylation, epigenetic alterations, and posttranslational modifications, which weaken the immune response and increase the risk of cancer [50].

Apart from the direct pathological effect, an absence of the appropriate microbial composition in the immune system's early development has more far-reaching effects. This is evident from studies on mice reared in germ-free (GF) conditions. These animals have impaired GI-driven immune development, characterized by smaller Peyer's patches, fewer CD8*αβ* intraepithelial lymphocytes, underdeveloped isolated lymphoid follicles, a lack of primed T cells, lower levels and impaired production of mucosal IgA antibodies, and active IL-10-mediated inflammatory hyporesponsiveness [51, 52]. Also, mice with colitis-associated cancer (CAC) that lacked microbiota were unable to process pro-IL-1*β* and pro-IL-18 (interleukins in this case necessary for a desirable inflammatory reaction) into their mature forms, resulting in increased tumour burden [53]. That said, we can see that the composition of “healthy” microbiota is crucial to the proper development of the immune system's basic structures. Unless fully developed, their ability to exchange information and their reactions to the “outside” world are compromised. This state could be characterized as similar to anergy: the signal is present but the immune system does not respond, as exemplified in the case of CAC mice.

## 3. Lung Microbiota

The human respiratory tract is the primary and continuous entry portal for numerous microorganisms and particles, such as viruses, bacteria, or fungi. These are primarily airborne but can also be transferred through saliva. Below the vocal cords, the human airways harbour bacteria and other microbes in rich surroundings [54] that are distinct in composition from the microbiota seen at other sites (in the nasal and oral cavity, gut, skin, and vagina). Despite being less populated compared to the GI tract, the lung microbiota includes a range of microorganisms, mostly seen through the use of bronchoalveolar lavage (BAL) fluid, or tissue samples. Since lung microbiota exploration has rather young and nonuniform protocols, it is crucial to bear in mind that the type of sample (lavage, tissue), sampling method, and possibility of cross-contamination during sampling between distinct parts of the airway influence final results [19, 55]. Therefore, due to the paucity of overall studies in this field, one must be careful to take detailed methodology and its possible advantages and disadvantages into account. Studies that analyse lung tissue acquired through sterile surgical explant have also been carried out, and they all report that the lower respiratory tract contains a microbiome that is distinct from, but related to, that of the upper airways [56].

In 2014 Dickson et al. [57] proposed an adapted island model for lung biogeography, suggesting that more distal lung bacterial communities are less rich and more dissimilar to their upper respiratory tract source community. Here, microbial composition is determined by the rate of microbial immigration into the airways, their rate of elimination (e.g., by coughing or immune defences), and the rate at which different community members are reproduced [58]. Temperature, oxygen tension, pH, nutrient density, local anatomy, and host defence are spatially heterogeneous across the airways and the lungs, all of which affect local microbiological growth conditions.

Starting with the upper respiratory airways, the nostril is dominated by Firmicutes and Actinobacteria; Firmicutes, Proteobacteria, and Bacteroidetes are prevalent in the oropharynx [13]. In the lung the most common phyla consistently observed are Bacteroidetes, Firmicutes, and Proteobacteria. The nasal microbiota seems to more closely resemble that of the skin than that of the lungs and contributes little to lung communities [59]. The coming years are likely to bring the development of new methods able to minimize cross-contamination (as seen for endoscopy [60]), which is the biggest problem in lower respiratory tract sampling that, along with metagenomic analysis (of both cultivable and noncultivable bacteria), will yield more precise results in terms of the bacterial population found at certain airway depths. An overview of different studies investigating the composition of the respiratory system microbiota is presented in Table 1.

The ecological determinants of the lung microbiota (immigration, elimination, and regional growth conditions) change during acute and chronic lung disease, as seen in chronic obstructive pulmonary disease (COPD) (often a precancerous inflammatory state) and lung cancer (Table 2) [7, 19]. Whether the observed dysbiosis is a cause, consequence, or simply a coevolving factor still needs to be elucidated, but its likely role will be individually connected to the pathology and its aetiology. However, it is known that smouldering inflammation (caused by lung injury, pathogen colonization, or intrinsic factors) is often a common starting point that leads to subsequent cancer development [61].

The microbial factors that may be responsible for lung cancer development are still not well known, unlike the many genetic predispositions and mutations that underlie the different types of lung cancer [62]. This is why, for now, it is possible to correlate the nongenetic development of lung cancer with the well-characterized development of COPD, a chronic inflammatory state where initial lung injury, whatever its cause, creates an opening for microbial dysbiosis and colonization, thus worsening the overall condition and often leading to the cancer state.

Inflammation of the lung is associated with a loss of epithelial integrity and results in the “leakage” of serum proteins into the airways [63]. Formyl peptides and cleavage products of bacterial or mitochondrial proteins, as well as other bacterial products, serve as powerful chemoattractants for both the neutrophils and monocytes that emigrate from alveoli [64]. Although essential in pathogen clearance and having a tumoricidal effect [65], neutrophil influx and degranulation in the airways and lung parenchyma contribute to chronic inflammation, parenchymal lung damage, and progressive small airway obstruction, due to the loss of alveolar attachments and lung elasticity [66], as explained by Sethi's vicious circle hypothesis [67].* In vitro*, their enzymes, serine proteinases (elastase, cathepsin G, and proteinase 3), and defensins markedly affect the integrity of the epithelial layer, decreasing the frequency of the ciliary beat, increasing mucus secretion, and inducing the synthesis of epithelium-derived mediators such as neutrophil chemoattractant chemokine IL-8 from respiratory epithelial cells [68].

Obstruction of the lumina with mucus introduces pockets of increased temperature and decreased oxygen tension, selectively favouring the growth of well-known disease-associated microbes [69, 70]. This dysbiotic shift can be characterized by a move away from the Bacteroidetes phylum, often to Proteobacteria (e.g.,* Pseudomonas aeruginosa*,* Haemophilus influenza, *and* Moraxella catarrhalis*) [17, 20], and sometimes to Firmicutes (e.g.,* Streptococcus pneumoniae *and* Staphylococcus aureus*) [19]. The same growth effect was observed with a generation of intra-alveolar catecholamines and inflammatory cytokines [71]. If airway colonization becomes persistent, it further promotes chronic inflammatory response, affecting the elastase-antielastase balance in the lung, which was shown to vary 80-fold with changes to the airway bacterial load [72]. A higher bacterial load consequently induces higher overall IL-8 and other blood circulating inflammatory cytokine levels, greater inflammation, oxidative stress, and greater forced expiration volume (FEV) decline [73].

Lung microbiota was also shown to vary according to clinical endpoints. In nonmalignant lung tissue from advanced stage cancer, alpha diversity had increased, while it had decreased in the tumour lung tissue. Also, the interaction between the upper and lower airways involving the microbial population present in lung cancer is clearly shown in the study carried out by Yan et al. [21]. The study showed that there is a high degree of specificity in patients with either small cell carcinoma (SCC) or adenocarcinoma (AC), compared to controls based on bacteria isolated from saliva (Table 2).

As mentioned above, IL-6 and IL-8 are cytokines that become elevated during inflammatory stress. They are involved in tumorigenesis by acting directly on lung epithelial cells to stimulate the NF-*κ*B-1 pathway [74]. Additionally, IL-6 and IL-8 are expressed by premalignant or senescent lung cancer cells [75]. They may act in an autocrine and/or paracrine fashion to stimulate cancer cell proliferation [76], migration, and invasion [77]. In bronchoalveolar carcinoma, tumour cells were a main source of IL-8 and the presence of an increased number of neutrophils in BAL fluid was correlated with the IL-8 level in BAL and associated with a poor outcome [78]. In a case study carried out by Pine et al. [79], increased levels of both serum IL-6 and IL-8 were associated with lung cancer, but only the IL-8 level was associated with lung cancer risk several years prior to diagnosis.

To summarize, the appearance of dysbiosis or malignancy is likely the product of a dynamic interaction between various immune, microbial, and environmental factors. At least one of these acts as an initiator but others often readily follow. This is why it remains difficult to reach any conclusions regarding the true aetiology of disease and what might be the best intervention and, more importantly, prevention approach.

## 4. A Bidirectional Concept of the Gut-Lung Axis

Recently, we have reached a greater understanding of microbial influence on the complex and interconnected axis between gut and lung. This stems from the simple fact that ingested microorganisms can access both sites—from gastrointestinal tract microbiota that enters the lung through aspiration [80] to the more “internal” influence, which shows improved lung function and pathogen clearance following the transplantation of faecal microbiota [6].

**Figure 2:**
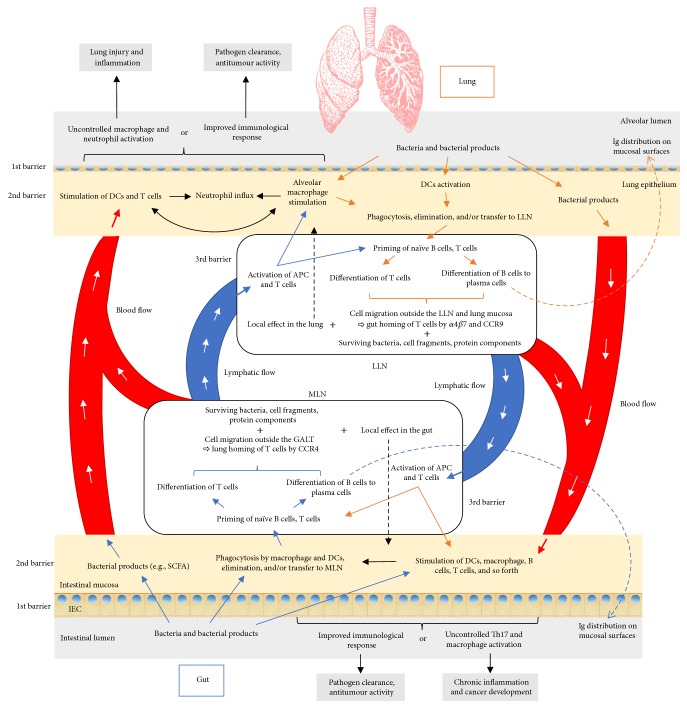
Proposed pathways of the gut-lung interaction. Microbiota and its products that enter intestinal mucosa (blue arrows) are phagocytosed and transferred to mesenteric lymph nodes (MLN) by antigen presenting cells (APC), where they stimulate priming of the T and B cells. Once activated, with the expression of proper homing receptors, these cells can migrate back to the original site (intestinal mucosa) (black dashed arrow) or to distal locations such as the lung epithelium and lung nodes through lymphatic and blood circulation. There, they can directly act on their target or continue to stimulate other immune cells. On the other hand, bacterial products from the intestinal mucosa or surviving bacteria can also reach the lung by blood or lymphatics to stimulate the immune system in the same way as they would have done in the intestinal tract. Depending on the tissue prestimulation, type of stimulus, and local and general immunological status, the result can be positive, as effective bacterial clearance or antitumour activity, or overinflammatory response, promoting further tissue damage, pathogen colonization, and tumour progression. The same schema is proposed in the other sense, beginning with the lung mucosa and finishing with distal effect on the gut. Although not known for the moment, there is also a possibility that bacterial products of the lung microbiota can exert their effect in the intestinal mucosa, being delivered in the same way as explained above. APC: antigen presenting cell; DC: dendritic cell; GALT: gut-associated lymphatic tissue; IEC: intestinal epithelial cell; LLN: lung lymph node; MLN: mesenteric lymph node; SCFA: short-chain fatty acid. Colour legend (borders/arrows): blue: influence of gut on lung; orange: influence of lung on gut; black: mutual influence.

This interaction can be mediated in different ways—by the microbiota and its products or via immune cells (Figure 2). According to the “gut-lymph” theory of Samuelson et al. [26], there are sufficient macrophages and other immune cells in the intestinal submucosa or the mesenteric lymph nodes that contain a majority of translocating bacteria. Surviving bacteria, cell wall fragments, or the protein parts of dead bacteria escaping with the cytokines and chemokines produced in the gut travel along the mesenteric lymphatic system to the cisterna chyli and subsequently enter the systemic circulation. Access to pulmonary circulation may lead to DC and macrophage activation as well as the priming of T cells and their differentiation.

Another way to influence the pulmonary region is through the migration of immunological cells. As previously mentioned, translocated microorganisms and their parts within the lamina propria are transferred to the mesenteric lymph nodes by antigen presenting cells (APCs) and used for priming naïve B and T cells. Activated B cells capable of producing antigen-specific immunoglobulins, that is, plasma cells, will not only produce immunoglobulins in situ, but will reach draining lymph nodes and other mucosal tissues, thereby spreading immunological “information.” The constitutive entry of antigen at steady state stimulates inflammasome conversion of pro-IL-1*β* and pro-IL-18 into active form; in other words it switches off our innate ability to produce IL-10 and other anti-inflammatory molecules leading to DC migration to local lymph nodes and the priming and differentiation of T cells. The latter can subsequently migrate out of the gut-associated lymphatic tissue (GALT) and reach both mucosal and peripheral nonmucosal tissues, including the bronchial epithelium, thus modifying the immunological response which is dependent on the induced cell profile (to Th1, Th2, etc.) [81, 82] and improving the immunological response against pulmonary pathogens [83].

Although this theory explains the unilateral interaction, it is reasonable to speculate that this axis works in precisely the same way when it originates in the lung mucosa and lung lymph nodes (Figure 2). Further, lung DCs* in vitro *have the option to imprint the expression of gut-homing integrin *α*4*β*7 and CCR9 (lung-homing integrin is CCR4 [84]) on cocultured T cells* in vitro* and on adoptively transferred cells* in vivo*, which guides their migration to the GI tract [85].

### 4.1. Influence of the Gut Microbiota on the Lung

The composition of “healthy,” or rather balanced, gut microbiota is shown to have a serious influence on the effectiveness of lung immunity. GF mice, devoid of their intestinal microbiota during the development of their immune system, show impaired pathogen clearance in the lung, which results in their growth and dissemination [52]. At this stage, it is also important to consider that the lungs of these mice are also germ-free [86], devoid of all microbiota that might normally play a role in stimulating lung immunity. Modified alveolar architecture also results, and thus both factors modify the response to pathogen infection. The same observations were made as with the infection of GF animals, when wild phenotypes were treated with antibiotics, thus disturbing the intestinal homeostasis, after they had been challenged with bacterial or viral microorganisms [87, 88]. If animals had been boosted with lipopolysaccharide (LPS) following antibiotic treatment, they were better able to cope with the lung infection and consequently had reduced mortality. Population studies followed these findings with respect to the importance of preserving gut microbiota, showing that increased use of penicillins, cephalosporins, macrolides, and quinolones correlated with an increased risk of lung cancer in humans [89]. Here we can speculate that various antibiotic treatments eradicate the bacterial populations required for effectively priming T lymphocytes with antitumour properties, while at the same time making room for other opportunistic pathogens to colonize both the gut and the lung.

Interestingly, modified gut microbiota not necessarily characterized as dysbiotic may also influence immune response efficiency, as seen in obese mice. These mice had an impaired expression of cytokines in their lungs (IFN*α*, IFN*β*, IL-6, and TNF*α*) and significantly decreased mRNA of IFN*γ*, interleukin 2 receptor subunit beta (IL-2RB), and perforin 1 (Prf1). All the criteria were improved following daily supplementation with a probiotic strain of* Lactobacillus gasseri *[90].

Nutrition may also impact microbial development and the composition of our respiratory tract microbiota [91]. A high-fibre diet in mice has been shown to increase SCFA circulating blood levels (but no traces in the lung itself) and has been shown to be responsible for increased protection against allergic inflammation in the lung (reduced inflammatory cell infiltration), followed by a change in the intestinal and, to a lesser extent, the airway microbiota [92].

The above-mentioned findings clearly show how important the overall composition of the intestinal microbiota is for a productive immunological response in the lung. Lack of an appropriate stimulus during the developmental phase, as during infection, will disable a quick and effective immune reaction, resulting in pathogenic colonization, increased susceptibility to infection, damage, the possible development of cancer, and increased mortality. At the same time, just one single strain, bacterial part, or product can turn the tables and provide the boost needed to stimulate the correct immune response.

### 4.2. Influence of the Lung Microbiota on the Gut

Unlike the local and systemic influence of intestinal microbiota, the influence of lung microbiota and its products and their circulation is yet to be properly assessed. One study reported that nonabsorbable tracer deposited into the nasal cavity of mice can be found in the GI tract a short time later [93]. Also, Sze et al. [54] showed that, in mice, even acute exposure to a single dose of intratracheal LPS disrupts the airway microbiota, leading to translocation of these bacteria into the bloodstream. Within 24 hours the caecal microbiota is also disturbed, leading to a sharp increase in the total bacterial load. It still remains unclear as to whether this effect is due to the direct interaction of translocated pulmonary and residential intestinal microbiota via immune cell or cytokine mediation or to microbial products that reach the gut.

### 4.3. Microbiota and Cancer via the Immune System

Due to a number of genetic alterations resulting in the loss of normal cellular regulatory processes, cancer cells express neoantigens that are tumour-specific and distinguish tumour cells from healthy cells. The importance of the gut microbiota in anticancer response has been described by Chen and Mellman [94] through the concept of a cancer-immunity cycle. The cancer-immunity cycle starts with the capture of neoantigens from cancer cells by DCs. For an anticancer response to take place, this must be accompanied by another signal, such as proinflammatory cytokines, factors released by dying cancer cells or by gut microbiota components. The goal here is to reduce peripheral tolerance to tumour antigens. Following processing, DCs present captured neoantigens to T cells, thus resulting in their priming and activation to create effector T cells against cancer-specific antigens. At this point, the balance between T effector and T regulatory cells is crucial in determining the nature of the immune response. The now activated T effector cells travel to the tumour site, invade the tumour bed, and, by recognizing specific tumour antigens, bind and kill cancer cells. Problems arise when tumour antigens are not detected, meaning that DCs and T cells treat antigens as “self” rather than foreign. In this case, a Treg response rather than an effector response results. Homing of T cells to tumour may not be correct either. T cells may be inhibited from infiltrating the tumour, or (more importantly) factors in the tumour microenvironment may suppress any effector cells that are produced. There are two main negative regulators of T cell responses: checkpoints in lymphoid organs (CTLA-4) and immunostats within the tumour beds (PD-L1:PD-1). Programmed cell death ligand 1 (PD-L1) is a molecule expressed on tumour cells or on tumour-infiltrating immune cells, which binds programmed cell death protein 1 (PD-1) expressed on effector CD8^+^ T lymphocytes, blocking the secretion or production of the cytotoxic mediators needed to kill tumour cells within the tumour beds. Cytotoxic T-lymphocyte-associated protein 4 (CTLA-4), expressed on Tregs, acts as the major negative regulator of the priming and activation of effector CD8^+^ T cells inside the lymphoid organs, by binding its CD80 and CD86 ligands on APCs. The presence of these suppressive factors explains the limited activity of previous immune-based therapies. The goal of current cancer immunotherapy, using anti-PD-L1:PD-1 and anti-CTLA-4 antibodies, is to initiate or reinitiate a self-sustaining cycle of cancer immunity, enabling it to amplify and propagate without creating an unrestrained response.

To create a higher response to neoantigens, the immune system's peripheral tolerance must be reduced. It is already known that the commensal microbiota induces the generation of CD4^+^ T cells against their own antigens [95] thereby limiting the systemic dissemination of commensal bacteria [96]. The same antigen cross-reactivity effect, or superantigen-driven response, accounts for T cell-dependent tumour regression. As suggested by Viaud et al. [47] and based on recent studies by Iida et al. [97] and Viaud et al. [98] in mice, Th17 cells and memory Th1 cells elicited against commensal bacteria might preferentially accumulate in an inflammatory tumour microenvironment, already primed by bacterial products or ligands for PRRs. Based on these studies, Zitvogel et al. [99] explain the long-range effect of microbiota through two signal hypotheses. Signal 1 hypothesis suggests a phenomenon of antigen mimicry or cross-reactivity. That is, certain microbial antigens from the bacterial species that pass the intestinal barrier and are used for T cell priming could closely resemble tumour antigens, thus promoting better immune system reactivity and antitumour response, that is, immunosurveillance. In signal 2 hypothesis, by interacting with PRRs after passing the intestinal barrier, microbiota can stimulate the production of a diverse palette of cytokines and interferons and determine whether it will elicit a proinflammatory, immunostimulatory, or immunosuppressive response. Also, there is some evidence that commensal-specific Tregs are capable of switching to effector inflammatory Th17 cells after sensing the disruption of the mucosal barrier. Because microbial products, metabolites, effector cells, and cytokines are able to travel, this stimulation is not necessarily confined to just the gut.

Observing these effects, it is interesting to speculate that at least a transient disruption of intestinal barrier functions and microbiota translocation is a primary factor in shaping the relationship between the gut microbiome, the immune system, and cancer.

### 4.4. Probiotics and the Lung

Probiotics, best known in nutritional therapy, are defined as “live microorganisms, which, when administered in adequate amounts, confer a health benefit on the host” [100]. In the intestine they mainly refer to the genera* Lactobacillus *and* Bifidobacterium* and include many different strains such as* L. paracasei*,* L. rhamnosus*,* L. acidophilus*,* L. johnsonii*,* L. fermentum*,* L. reuteri*,* L. plantarum*,* B. longum, B. breve, B. bifidum, *and* B. animalis *subsp.* lactis*. The same genus does not necessarily involve the same characteristics, due to the great genomic differences between species and also within the different strains of the same species [101].

Although the first evidence of probiotic influence on lung cancer was seen in 1985 [102], probiotics only recently reemerged in the field of lung oncology as a possible new therapy and is already showing highly promising results. Using different mouse lung cancer models, conventional therapies (a combination of platinum-based agents with paclitaxel, gemcitabine, vinorelbine, or docetaxel, which all have high toxicity [103]) were combined with specific probiotic strains or, conversely, the antibiotic eradication of microbiota, to assess the effect of chemotherapy.

In a lung adenocarcinoma viral model, when vancomycin was used to eradicate Gram-positive bacteria it compromised the efficacy of cyclophosphamide- (CTX-) based chemotherapy and correlated with a reduced intratumoural CD8^+^ T effector/FoxP3^+^ regulatory T cell ratio [104]. Likewise, in mice treated with cisplatin combined with an antibiotic cocktail of vancomycin, ampicillin, and neomycin, tumour size was larger than that found in mice receiving a single treatment of cisplatin [105]. Taking these examples, we can readily conclude that the presence of conventional intact microbiota is crucial for effective chemotherapy.

On the other hand, feeding mice orally with* L. acidophilus* (Lewis lung cancer model) treated with cisplatin decreases the size of tumours and improves the survival rate. Enhanced antitumour response is also achieved through upregulation of IFN*γ*, granzyme B (GzmB), and Prf1 expression [105] following probiotic supplementation.

Recently, there was considerable interest in evaluating the role of gut microbiota in lung cancer therapy using immune checkpoint inhibitors. One of the first studies of this principle was done using a mouse melanoma model but is readily applicable to other cancer types, as shown in the study. Here, oral administration of a* Bifidobacterium *cocktail (*B. bifidum, B. longum, B. lactis, *and* B. breve*) on its own improved tumour control to the same degree as PD-L1*-*specific antibody therapy (checkpoint blockade) [106]. When the two treatments were combined, it virtually abolished tumour outgrowth. Improvement was seen in immune responses upstream of T cells, at the level of host DCs. Their augmented function enhanced CD8^+^ T cell priming and accumulation in the tumour microenvironment. The percentage of MHC II^hi^ DCs was also increased. With* Bifidobacterium *treatment, 760 genes were upregulated, including cytokine-cytokine receptor interaction, CD8^+^ T cell activation and costimulation, DC maturation, antigen processing and cross presentation, the chemokine-mediated recruitment of immune cells to the tumour microenvironment, and type I interferon signalling.

Vétizou et al. [107] reported that the oral feeding of GF mice with* Bacteroides fragilis *induced Th1 immune responses in tumour-draining lymph nodes and promoted the maturation of intratumoural DCs. This was, as the authors suggest, due to the cross-reactivity of the bacterial and tumour epitopes, which led to the restoration of the therapeutic response of GF tumour bearers to CTLA-4 antibody treatment. Antibody therapies such as this prevent inactivation of the CD8^+^ T cells by binding to the CTLA-4. Daillère et al. [108] noted that* Enterococcus hirae* and* Barnesiella intestinihominis* specific-memory Th1 cell immune responses selectively predicted longer progression-free survival in advanced lung cancer patients treated with chemoimmunotherapy. Both strains represent valuable “oncomicrobiotics" improving the efficacy of the most common alkylating immunomodulatory compound.

To summarize, as scientists delve deeper, the beneficial effects of probiotics on the immune system continue to emerge. As seen, certain strains have the power and ability to stimulate antitumour response or to simply stimulate the immune system to show lower tolerance, thus promoting higher reactivity and tumour eradication. The future objective is to find the optimum probiotic cocktail that may one day completely substitute conventional therapies, thereby obtaining equal or better success and lowering toxicity, one of the biggest problems in cancer treatment.

## 5. Conclusion

The importance of the gut microbiota and its composition has long been recognized, for digestion as well as for overall wellbeing. Recently, the presence of the lung microbiota and the role it plays, in health and in disease, have been receiving attention. The lung and gut microbiota, both continually reseeded through interaction with the environment, modulate our local and systemic immunity. More than simply two distinct microbiota, they are now seen as functioning in dialogue, altering previous ideas of airway sterility and the existence of a “barrier” between the two compartments, due to their perceived distance or functional differences. By providing stimulating signals through its epitopes or products (such as SCFA butyrate), the gut microbiota directly enhances the intestinal barrier. Likewise, it stimulates the priming and maturation of T and B cells, ensuring improved microbial clearance and mucosal protection through antibodies. This effect is not only retained in the intestinal system but is spread along other mucosal surfaces by means of lymphatic and blood circulation, influencing distal site immune response. So, even though the antigen was introduced in the gut, an immunological response can also be elicited in the lung, although there was no direct prior contact with the antigen, and vice versa. Bacteria and their products that go through the first immunological barrier also reach distal sites through the lymphatic system and blood and modulate the immune response at the remote site. The site where the first encounter between the immune system and microbial antigens took place is also important, since it influences reactivity and the influx of these cells into other tissues. Applying prebiotics to target a specific microbial group could be a good way to restore “healthy” microbial composition, which will consequently increase intestinal barrier function and stimulate the immune system. The relevance of natural microbial support in chemotherapy effectiveness or replacement has already been demonstrated. In future, further discoveries will surely be made in this new and exciting area of research, adding to the complexity of, but also clarifying the reasons behind, this axis; opening ideas to new or enhanced therapies based on the natural behaviour of the organism; increasing longevity; and decreasing therapeutic side-effects or the effects of disease itself.

## Figures and Tables

**Table 1:** Most frequently detected bacteria in GI tract and respiratory system of healthy volunteers or from healthy tissue samples. Results from different studies are presented by the taxa level in which they were originally detected, in order of decreasing abundance where possible. If the sampling, analysis method, or result was specific for a certain study, the reference was added adjacent to the corresponding information.

	Sample source Analysis method	Phylum	Order or family	Genus or species	Reference
GI tract	Faeces qPCR^a^, 16S sequencing^b^	Firmicutes(79.4% of sequences), Bacteroidetes (16.9%), Actinobacteria (2.5%), Proteobacteria (1%), and Verrucomicrobia (0.1%)^b^		*Faecalibacterium, Ruminococcus, Eubacterium, Dorea, Bacteroides, Alistipes, *and* Bifidobacterium*^a^ *Bacteroides (vulgatus), Roseburia (intestinalis), Ruminococcus (bromii), Eubacterium (rectale), Coprobacillus *(sp.),* Bifidobacterium (longum), *and* Clostridium (leptum, coccoides)*^b^	Tap et al. [11]

Oral cavity	Saliva 16S sequencing	Firmicutes, Proteobacteria, Actinobacteria, Fusobacteria, TM7, and Spirochaetes	Pasteurellaceae (5.8%), Enterococcaceae (2.6%), Veillonellaceae (2.0%), Burkholderiales (1.2%), and Lactobacillales (1.1%)	*Neisseria* and *Streptococcus* (70% of sequences), *Haemophilus* (9.2%), *Leptotrichia* (2.1%), *Actinomyces* (1.9%), *Abiotrophia* (1.8%), *Atopobium* (1.6%), *Gemella* (1.6%), and *Arthrobacter* (1.0%)	Lazarevic et al. [12]

Nose	Swab 16S sequencing	Actinobacteria, Firmicutes, Proteobacteria, Bacteroidetes, and Fusobacteria	Staphylococcaceae, Lachnospiraceae [13] Propionibacteriaceae, Corynebacteriaceae [14]		Charlson et al. [14] Lemon et al. [13]

Oropharynx	Swab 16S sequencing	Dominated by Firmicutes, Proteobacteria, and Bacteroidetes, and Fusobacteria, Actinobacteria, TM7, and SR1 follow (oropharynx was richer and less variable than the nostril microbiota)	Streptococcaceae, Lachnospiraceae, unclassified group of Clostridia [13] Streptococcaceae, Veillonellaceae, Fusobacteriaceae, and Neisseriaceae [14]		Charlson et al. [14] Lemon et al. [13]

Esophagus	Biopsy 16S sequencing	Firmicutes, Bacteroidetes, Actinobacteria, Proteobacteria, Fusobacteria, and TM7		*Streptococcus (mitis, thermophiles, parasanguis), Prevotella (pallens), Veillonella (atypica, dispar), Rothia (mucilaginosus), Megasphaera* (micronuciformis), *Granulicatella (adiacens), Gemella, TM7, Actinomyces (odontolyticus), Bacteroides, Clostridium, Haemophilus, *and* Bulleidia (moorei)*	Pei et al. [15]

Lung	BAL^a^, brushing^b^ 16S sequencing	Actinobacteria, Firmicutes, and Proteobacteria^a^ [16] Bacteroidetes, Firmicutes, Proteobacteria, Actinobacteria, andFusobacteria^b^ [7]	Streptococcaceae, Veillonellaceae, Prevotellaceae, Micrococcaceae, Neisseriaceae, Porphyromonadaceae, Lachnospiraceae, Actinomycetaceae, and Fusobacteriaceae^a^ [14]	*Pseudomonas, Streptococcus, Prevotella, Fusobacterium, Haemophilus, Veillonella, *and* Porphyromonas*^a^ [17] *Prevotella, Veillonella*, other *Firmicutes*, other *Bacteroidetes*, *Streptococcus, Haemophilus, Neisseria, Fusobacterium, *other* Actinobacteria, Staphylococcus, *other* Proteobacteria, *and* Corynebacterium*^b^ [7]	Charlson et al. [14] Erb-Downward et al. [17] Hilty et al. [7] Pragman et al. [16]

BAL: bronchoalveolar lavage.

**Table 2:** Most frequently detected bacteria in the lung of patients suffering from their respective diseases. Results from different studies were presented by the taxa level in which they were originally detected, in order of decreasing abundance where possible. If the sampling, analysis method, or result was specific for a certain study, the reference was added adjacent to the corresponding information.

	Disease	Sample source	Phylum	Order or family	Genus or species	Reference
		Analysis method				
Lung	COPD	BAL^a^, sputum^b^, lung tissue^c^ 16S sequencing^d^, qPCR/DGGE^e^, bacterial culture^f^	Proteobacteria (44%) and Firmicutes (16%) followed by Actinobacteria (13%), with Bacteroidetes,Fusobacteria, Tenericutes,SR1 incertae, TM7, and Synergistetes identified in lower proportions (<3%)^b,d^ [18] Increase in Proteobacteria(sometimes Firmicutes), decrease in Bacteroidetesand Firmicutes^c,d^ [19] In moderate COPD mostly Actinobacteria and Proteobacteria In severe COPD mostly Actinobacteria and Firmicutes^a,d^ [16] Proteobacteria, Bacteroidetes, Firmicutes, Actinobacteria, and Fusobacteria^b,d^ [7]		*Haemophilus influenza, Pseudomonas aeruginosa, Moraxella catarrhalis, *and* Streptococcus pneumoniae*^b,f^ [18] Significant increases of* Streptococcus pneumoniae, Klebsiella pneumoniae, *and* Pseudomonas aeruginosa*^b,e^ [20] *Streptococcus, Abiotrophia, Rothia, Tropheryma, Actinomyces, Peptostreptococcus, Serratia, Capnocytophaga, Leptotrichia, Kingella, *and* Dysgonomonas*^a,d^ [16] Significant abundance of *Pseudomonas *and *Haemophilus*^a,d^ [17] *Prevotella, Haemophilus, Streptococcus, Neisseria*, other *Proteobacteria, Veillonella*, other *Firmicutes*, other *Bacteroidetes*, *Fusobacterium*, other *Actinobacteria*, and *Staphylococcus*^b,d^ [7]	Erb-Downward et al. [17] Garcia-Nuñez et al. [18] Hilty et al. [7] Sze et al. [19] Wu et al. [20] Pragman et al. [16]
	Lung cancer	Sputum^a^, saliva^b^ anaerobic culture^c^, 16S sequencing^d^		Flavobacteriales, Burkholderiales, Campylobacterales, Spirochaetales(more abundant), and Bacteroidales(less abundant)^b,d^ [21] Veillonellaceae significantly overexpressed whereas Lachnospiraceae underexpressed^b,d^ [21]	*Actinomyces* spp., *Peptostreptococcus* spp., *Eubacterium lentum, Veillonella parvula, Prevotella* spp., *Bacteroides* spp., *Lactobacillus jensenii*^a,c^ [22] *Capnocytophaga, Selenomonas, *and *Veillonella *were found to be more abundant in both SCC and AC^b,d^ [21]	Rybojad et al. [22] Yan et al. [21]

COPD: chronic obstructive pulmonary disease; BAL: bronchoalveolar lavage; DGGE: PCR-denaturing gradient gel electrophoresis; SCC: small cell carcinoma; AC: adenocarcinoma.
